# Epidemiological and Clinical Evidence for the Role of Toxins in *S. aureus* Human Disease

**DOI:** 10.3390/toxins12060408

**Published:** 2020-06-19

**Authors:** Monique R. Bennett, Isaac P. Thomsen

**Affiliations:** 1 Department of Pediatrics, Vanderbilt University School of Medicine, Nashville, TN 37232, USA; monique.r.bennett.1@vumc.org; 2 Vanderbilt Vaccine Research Program, Nashville, TN 37232, USA; 3 Vanderbilt Institute for Infection, Immunology and Inflammation, Vanderbilt University Medical Center, Nashville, TN 37232, USA

**Keywords:** *Staphylococcus aureus*, toxins, vaccines, monoclonal antibodies

## Abstract

*Staphylococcus aureus* asymptomatically colonizes approximately 30–50% of the population and is a leading cause of bacteremia, bone/joint infections, and skin infections in the US. *S. aureus* has become a major public health threat due to antibiotic resistance and an increasing number of failed vaccine attempts. To develop new anti-staphylococcal preventive therapies, it will take a more thorough understanding of the current role *S. aureus* virulence factors play in contributing to human disease. This review focuses on the clinical association of individual toxins with *S. aureus* infection as well as attempted treatment options. Further understanding of these associations will increase understanding of toxins and their importance to *S. aureus* pathogenesis.

## 1. Introduction

*S. aureus* asymptomatically colonizes the anterior nares and skin of ~30% of the human population [1]. Though it may start as a commensal, *S. aureus* often becomes an invasive pathogen once it has breached normal host innate immune defenses and is capable of infecting nearly every organ in the body. *S. aureus* is a leading cause of an array of clinical syndromes, including skin and soft issue infections, endocarditis, and osteomyelitis [2]. In recent years, the increased incidence of community-associated methicillin-resistant *S. aureus* (MRSA) infections, along with the high potential severity of *S. aureus* infections in general, has led to the widespread use of antistaphylococcal antibiotics, resulting in a current paucity of effective treatment options for *S. aureus*. Although there are a number of antibiotics approved for the treatment of *S. aureus* infection, resistance rates continue to rise, and there are currently no licensed vaccines available to prevent or ameliorate *S. aureus* disease. This is due in large part to the numerous immune evasion and virulence factors that may be potential candidates for vaccine development. This review will focus on a subset of these factors, the staphylococcal toxins, and the role this diverse group of virulence factors plays in contributing to human disease during *S. aureus* infection.

## 2. Role of *S. aureus* Toxins in Human Disease

### 2.1. Hemolysin-α (Hla or α-Toxin)

**Figure 1:**
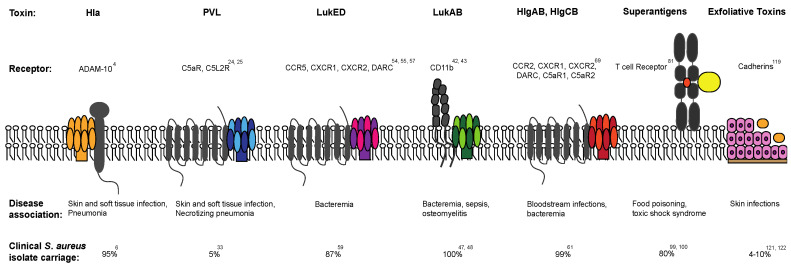
Receptors are now described for all of the clinically relevant toxins of *Staphylococcus aureus*. Disease associations are variable, as are reported rates of prevalence of specific toxin genes across clinical *S. aureus* isolates.

Hla is a pore-forming beta-barrel toxin, and one of the few *S. aureus* toxins that is core-encoded. This protein intoxicates multiple different cell types, including erythrocytes, T cells, and endothelial cells [3]. It often targets the cellular receptor ADAM10, a zinc-dependent metalloprotease, to bind to the surface of its target cell and initiate pore formation (Figure 1) [4]. The toxin in secreted as a soluble monomer, but, upon binding to ADAM10, the toxin oligomerizes and a pre-pore forms, followed by a transmembrane channel through the cell membrane [5].

Hla is secreted by approximately 95% of clinical *S. aureus* strains, with observable trends in distinct clonal types, such as an increase in Hla expression in CC398 strains, which correlates with stronger hemolysin activity [6]. Interestingly, CC398 is a commonly identified lineage of livestock-associated MRSA that can infect pigs as well as humans. A study analyzing LA-MRSA isolates from both pigs and humans was analyzed and compared to human HA- and CA-MRSA isolates. This study found that LA-MRSA isolates are highly cytotoxic in comparison with human HA- and CA-MRSA isolates but are less adherent to human cells than human isolates. All tested LA-MRSA CC398 strains also expressed *hla* when tested by qRT-PCR [6,7]. Hla has been notably associated with colonization of individuals who go on to develop invasive disease. In one study, an increase in expression of Hla between nasal colonization and bacteremia was noted [8]. Another study collected over 990 respiratory isolates from 34 countries and determined that *hla* was present in 99.6% of MSSA and 97.6% of MRSA isolates, underscoring its conservation [9]. A study in China characterized 47 clinical *S. aureus* isolates and found that 88% of the MRSA isolates had the same clonal background, CC8, and these strains also correlated to the *hla* genotype 1 or 2, although 25 *hla* genotypes were found in total [10]. These findings indicate that *hla* is both diverse and highly expressed throughout a number of populations.

The role of Hla in the pathogenesis of *S. aureus* disease has primarily been investigated in murine pneumonia and skin infection models. To investigate the importance of Hla in a murine pneumonia model, mice lacking the Hla receptor gene, *Adam10*, were constructed. ADAM10-/- mice were found to be resistant to lethal MRSA pneumonia in comparison to wild-type mice [11]. Further, knockout strains of Hla have also been investigated in pneumonia models and show no increase in disease burden in mice [12,13].

Data regarding the role of Hla in the pathogenesis of *S. aureus* infections in humans are less abundant, although evidence suggests that the toxin is at least expressed in the setting of human disease. For example, numerous studies have identified a robust and neutralizing antibody response to α-toxin [14,15,16] following sepsis or pneumonia. A pediatric study measuring antibody levels in 235 children found that antibody titers to Hla from convalescent sera correlated with protection against subsequent *S. aureus* infection up to a year later [17].

**Figure 2:**
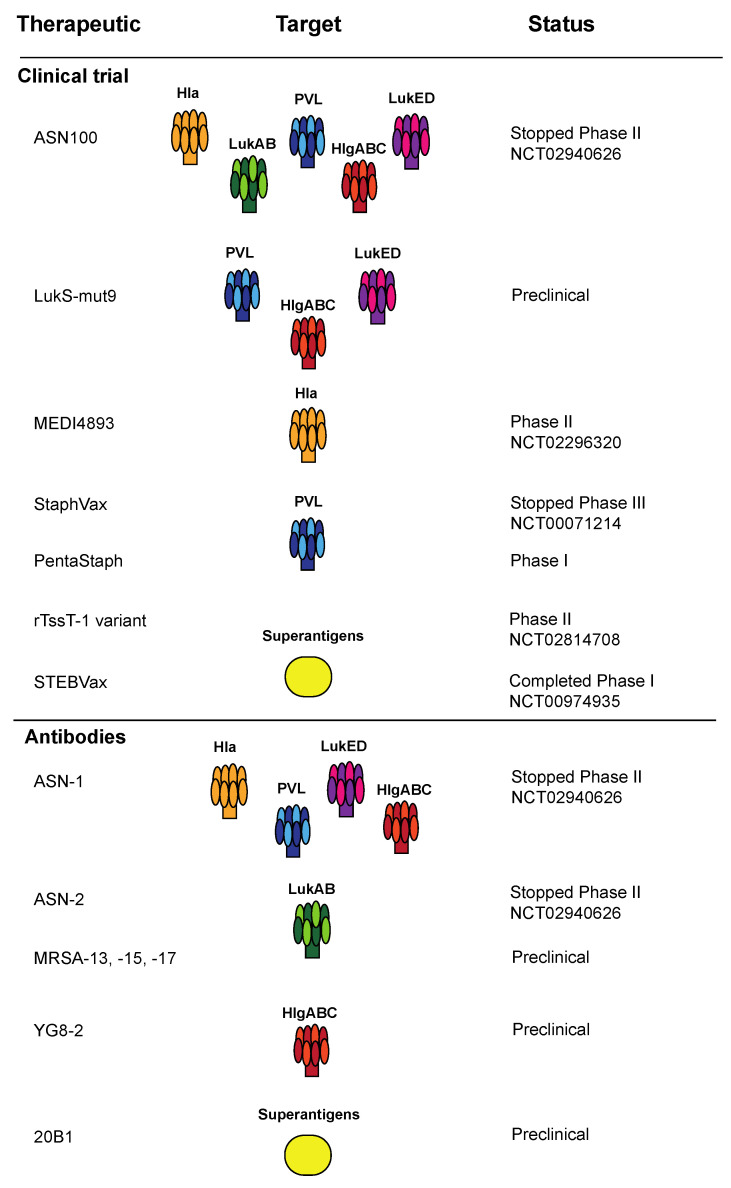
Prior reported attempts to intervene against *S. aureus* disease at the level of the host–toxin interaction, either by passive (e.g., monoclonal antibody preparations) or active vaccination.

Based on the strong evidence for its role in pathogenesis in murine models, along with its presumed importance in human disease as well, attempts to intervene against this toxin in the setting of human infection are ongoing. MEDI4893 is a monoclonal antibody capable of neutralizing Hla toxicity and preventing toxin-mediated platelet aggregation [18,19,20], one of the known complications of sepsis. A Phase 1 clinical trial of this antibody has been completed and found the antibody to be safely tolerated. Exploratory analyses of the Phase 1 study found evidence of detectable antibody for a prolonged period in serum and nasal washes following IV infusion [21,22]. The antibody has been further evaluated for efficacy as prophylaxis in a randomized phase 2 clinical trial of ventilated subjects with *S. aureus* infection, but this study has not yet been published (ClinicalTrials.gov identifier NCT02296320) (Figure 2). Other groups have also tested the efficacy of α-toxin for use in a vaccine. A mutated version of α-toxin, H35L, was shown to exhibit limited hemolytic activity [23]. The toxoid was tested in two different models and was shown to be effective through both active immunization and in the production of protective rabbit anti-Hla antibodies in a murine lethal pneumonia model [24], though this has not been assessed in human trials at the time of this writing.

### 2.2. Panton-Valentine Leukocidin (PVL)

PVL is a toxin composed of two monomers, LukS-PV and LukF-PV. PVL is the first of several leukocidins that will be considered, all of which target phagocytes, lymphocytes and natural killer cells. Chiefly important among the host immune cells targeted by leukocidins is the neutrophil. The leukocidins dimerize at the surface of the neutrophil and other phagocytes to form a pore that lyses and kills the cells. PVL targets the C5a receptor (Figure 1) [25,26], abundant on neutrophils, and has been shown to cause a number of different illness, including pneumonia, bacteremia, and necrotizing fasciitis, although it has been most notably linked with skin and soft tissue infections [27].

The causal role of PVL in human disease has been debated due to its geographical stratification and low circulating presence in clinical strains outside of community-associated (CA-) MRSA. PVL is most closely associated with CA-MRSA strains (~85%) [28], including the highly virulent USA300, sequence type 8 (ST8) in the United States [29], as well as the dominant CA-MRSA clone in Europe, North Africa, and the Middle East, ST80 [30]. Outside of these CA-MRSA clones, the observed frequency at which *pvl* is observed is highly variable. A cross-sectional study evaluating 300 athletes in France was unable to find any *S. aureus* isolates expressing PVL [31], while a study in Gambia analyzing 293 strains found that nearly 62% were PVL-SA positive [32]. A retrospective study in children analyzing the severity of PVL-SA found that, of 75 cases of PVL-SA infection, 10 developed severe disease, including necrotizing pneumonia, invasive soft tissue infection, and deep venous thrombosis, and the other 65 had superficial (e.g., cutaneous) infections [33]. Although PVL is associated with virulent community-associated strains, only about 5% of clinical strains overall contain the gene encoding the toxin, which undermines its clear contribution to *S. aureus* virulence [34].

Like many *S. aureus* toxins, PVL shows strong species tropism in favor of human neutrophils, which makes evaluating efficacy difficult in preclinical animal models. One study used humanized mice along with a *pvl* knockout strain to investigate the role of the toxin in vivo. The investigators found that PVL targets macrophages, with the Δ*pvl* strain having a 95% increase in overall macrophage numbers compared to WT-infected mice due to PVL toxicity. The Δ*pvl* strain also decreased bacterial burden compared to WT USA300 when tested during a pneumonia model [35]. These data are also consistent with previous animal data in rabbits that show the functional blocking of PVL by heavy-chain antibodies inhibits toxin-induced endophalmitis [36].

Studies indicate that PVL must be present at a certain concentration before cell lysis is effectively achieved in vivo [37,38]. When PVL is present at a lower threshold or sublytic concentration, PVL contributes to a number of proinflammatory pathways, including the release of IL-8 and IL-6, which can cause granulosis of polymorphonuclear leukocytes (PMNs) and the release of reactive oxygen species like O_2_^−^ [25,39]. PVL also leads to the release of IL-1β by macrophages, resulting in NLRP3 inflammasome activation [40]. The toxin also affects the behavior of PMNs, by causing an increase in CD11b on their cellular surface, priming them for increased bactericidal activity [39,41]. These findings indicate that PVL can alter cellular host responses by priming PMNs and altering their gene expression, without the need to cause cellular lysis.

Although early results in mouse models showed PVL subunits may make effective vaccines [42,43], these efforts have not successfully translated into human trials. Recombinant subunits of PVL were used in the development of the StaphVax vaccine (Nabi Biopharmaceuticals, Alpharetta, GA) [44]. While this vaccine was unsuccessful in phase 3 clinical trials, several antigens were repurposed, including PVL, into a new vaccine, PentaStaph, purchased by GSK (Figure 2). Aside from the use of monoclonal antibodies and vaccines, other clinical treatments have been studied when investigating the effectiveness of targeting toxins, including intravenous immunoglobulin (IVIg). IVIg consists of polyclonal antibodies that can be used to treat serious *S. aureus* infections. Commercial IVIg samples have been tested in several studies, and antibodies to PVL were confirmed by ELISA. IVIg treatment was further shown to protect rabbits from necrotizing pneumonia. Specific IVIg antibodies to both PVL and Hla were identified and found to be protective by prophylaxis in the necrotizing pneumonia model [45,46].

### 2.3. LukAB (Also Known as LukGH)

LukAB, also a bi-component leukotoxin, similarly targets neutrophils, monocytes, macrophages, and dendritic cells [47]. LukAB is comprised of two monomers, LukA (the S component) and LukB (the F component), which dimerize in solution before binding ( to the integrin, CD11b, at the surface of the target cell (Figure 1) [48,49]. The ability to dimerize before binding the cell is unique to LukAB, as other leukocidins oligomerize after the initial binding of the S component to the cell surface.

Due largely to species tropism favoring human leukocytes, assessing the importance of LukAB using disease models in animals has been a challenge. Initial studies testing the toxicity of LukAB by in vitro killing of polymorphic neutrophils using mice, rabbits, monkeys and humans noted the toxin had differential activity, with mice being the least susceptible. Similarly, when a LukAB knockout strain was tested in a skin model of infection in both rabbits and mice it did not lead to a decrease in disease burden [50]. However, initial studies performed in a murine renal abscess model showed a minor statistically significant decrease in bacterial burden when using a knockout strain of LukAB compared to WT mice [47]. These inconsistencies in animal models when studying LukAB have been traced to the strong tropism LukAB shows for human CD11b specifically [48,51,52]. Recently, the 11-base-pair domain within CD11b responsible for this species specificity was identified. With the use of CRISPR gene editing, this domain was used to create humanized mice, which showed an increased susceptibility to bacteremia when infected with USA300 [52]. Moving forward, this mouse model represents a prominent opportunity to define the role of LukAB in *S. aureus* pathogenesis with a clearer path to translation to human disease.

Expression of LukAB is highly conserved, with all clinical isolates tested containing the toxin [53,54]. Studies investigating the humoral response to LukAB have shown that antibodies to LukAB are functionally neutralizing and potent. This response has been consistent, leading investigators to develop a LukAB serologic assay capable of accurately distinguishing between individuals with and without *S. aureus* infection [55]. One study in children obtained acute and convalescent sera from over 50 children with invasive *S. aureus* infection and demonstrated an increase in both binding and toxin neutralization titer from the acute to the convalescent phase, consistent with the expression of LukAB in vivo during human disease [54]. The presence of LukAB antibodies has also been associated with specific diseases, such as cystic fibrosis, where an increase in LukA IgG titers in serum during pulmonary exacerbation with *S. aureus* infection has been reported [53]. Another study found an increase in anti-LukAB IgG-neutralizing titers in patients with bacteremic pneumonia [15]. In addition, functional monoclonal antibodies targeting LukAB have been isolated, using both yeast display and human hybridoma technology. In both cases, antibodies were able to functionally neutralize LukAB in phagocyte toxicity assays [56,57]. The human mAbs were shown to decrease bacterial burden in a murine septic model of infection, which underscores the important role LukAB plays in *S. aureus* disease [56]. In addition to the evaluation of monoclonal antibodies for their efficacy, IVIg has also been investigated. Evaluation of a variety of lots of commercially available IVIg showed that lots of IVIg contained antibodies that bound and neutralized LukAB, although the neutralizing capabilities varied between lots [58].

### 2.4. LukED

LukED, also a member of the bicomponent family of leucocidins, is comprised of the two secreted monomers LukE and LukD [59]. LukED specifically targets the chemokine receptors CCR5, CXCR1, and CDXCR2 on the surface of CCR5+ cells such as T cells, macrophages, and neutrophils (Figure 1) [60,61]. The specificity LukED shows for CCR5 has been investigated in detail. Monoclonal antibodies used in pull-down assays identified that CCR5 interacts with LukE specifically, although both components are necessary to mediate cellular toxicity. Interestingly, CCR5 is also a co-receptor for HIV, and the anti-HIV drug maraviroc is capable of blocking LukED-mediated cell death in human T-cell lymphoblast (HuT) cells, underscoring the importance of CCR5 in LukED-mediated cytotoxicity [60]. LukED is unique among the leukocidins in that it shows an increased tropism toward murine leukocytes. Studies indicate there is an increased potency towards mouse phagocytes using both in vitro and in vivo mouse models, including systemic and intravenous models [61,62,63,64].

While fewer assessments exist for LukED compared with the other leukotoxins, current epidemiological evidence indicates that toxin genes are common across *S. aureus* lineages, with ~87% of clinical isolates carrying *lukED* [65]. Studies done in China used PCR to investigate ~180 *S. aureus* isolates and found that between 81.4–92% of isolates contained *lukED* [66,67]. A similar percentage (82%) was found in blood isolates in a study done in Germany, although only about 61% of nasal isolates carried the locus [68]. Another study, spanning three continents and 117 CA-MRSA isolates, identified the *lukED* locus in 99% of the isolates, including HA-MRSA and CA-MRSA [69]. Nearly 300 isolates of USA400 collected and genotyped from the Midwest identified that *lukE* and *lukD* genes are found more often in CA-MRSA than clinical or nasal MSSA (~99%) [70]. LukED has been specifically associated with diabetic foot infection, where one particular study found the *lukED* locus in every MRSA isolate from a patient infected with a diabetic foot ulcer [71]. LukED has also been associated with the carriage of other virulence factors in *S. aureus* strains, including biofilm production in individuals with osteomyelitis [72].

While there are no current vaccines in clinical trials that include LukED as a primary antigenic component, preclinical studies have been performed on an attenuated subunit vaccine containing a mutated version of LukS-PV, LukS-mut9 (Figure 2). Because LukS-PV is cross-reactive with the S subunit of other leukocidins as well, polyclonal antibodies to LukS-PV bound and neutralized not only PVL, but LukED, as well as HlgABC [43,73].

### 2.5. γ-Hemolysins: HlgAB and HlgCB

Two γ-hemolysins are described, HlgAB and HlgCB, with *hlgA* and *hlgC* each encoding separate S subunits that combine with one F subunit, HlgB. HlgAB is noted for its ability to destroy erythrocytes, which has been observed in both rabbits and humans [74,75]. HlgAB binds the chemokine receptors CXCR1, CXCR2 and CCR2, while HlgCB targets the complement receptors C5aR and C5lA (Figure 1) [76]. The ability of HlgAB to target CCR2 is responsible for its specificity to murine neutrophils, which has been highlighted in a murine peritonitis model [76].

Studies that investigate the presence of *hlg* genes in circulating *S. aureus* strains have reported disparate results. When the virulence profiles of USA400 isolates were screened for differences between CA and HA-MRSA strains, no differential trend was detected for hemolysin genes [70]. Another study typed 334 invasive *S. aureus* isolates and found that, while *hlg* was present in most of the invasive isolates, there was no observable trend towards a particular disease type [77]. Similarly, a study in India reported that 98.2% of MRSA strains screened contained *hlg* [78] and another study in Iran found *hlg* present in all infected individuals when investigating isolates from nasal carriage [79]. In contrast, others have found lower rates of *hlg*, with one study in Iran reporting a frequency of only 6.3% in MRSA strains [80]. These widely divergent prevalence estimates may be a result of predominantly circulating clones in particular geographic regions, and further work is needed to gain a sense of the true prevalence of the g-hemolysins across *S. aureus* strains.

The contribution of γ-hemolysin to disease has been tested in a number of different animal models with conflicting results as well. The expression of *hlgABC* is clearly upregulated when *S. aureus* is cultured in human blood, consistent with its role in hemolysis [50]. When strains containing a knockout of *hlgABC* were tested using a murine skin and bacteremia model, only a minor reduction in disease was noted in the bacteremia model when comparing the knockout strain to wild-type MRSA. This indicates that HlgABC may be of limited importance in vivo [50]. Further, Hlg was shown to contribute to murine septic arthritis, but only when combined with α-toxin, further underscoring that it may contribute weakly to pathogenesis, possibly due to inherent redundancy in *S. aureus* virulence mechanisms [81]. HlgAB mutants have also been studied in a rabbit endophthalmitis model, wherein a Hlg-deficient strain was still capable of significant ocular infection, though with reduced inflammation of the eyelid [82,83].

Data regarding the potential role of the γ-hemolysins in human disease remain limited. Human monoclonal antibodies to HlgABC have been isolated using phage display technology. One mAb, YG8-2, was effective at reducing the leukocidin-mediated lysis of phagocytes and reduced bacterial burden in a peritonitis and bacteremia murine model of infection. Interestingly, however, YG8-2 was also cross-reactive with PVL and LukED [84]. The potential for cross-reactivity of a single monoclonal antibody across the leukocidins has been identified as a mechanism for putative intervention against this family of toxins. A single monoclonal, ASN-1, capable of neutralizing a-hemolysin plus four of the five leukocidins, was recently reported. Likely due to its more substantial structural differences, LukAB was not neutralized by ASN-1, and a separate antibody, ASN-2, was evaluated alongside ASN-1 as a bi-clonal approach to broad toxin neutralization (Figure 2) [62,85,86,87]. A single antibody capable of neutralizing all known leukocidins has yet to be reported.

### 2.6. Superantigens/Enterotoxins

*S. aureus* superantigens represent a large group of toxins capable of cross-linking T cell receptors with the major histocompatibility complex (MHC)-II molecules on antigen-presenting cells. Most superantigens bind specifically to the Vβ region of the T cell receptor, so that both the MHC class II and TCR are cross linked together (Figure 1), with the exception of the two superantigens, SEH and SEY, which bind instead to Vα [88,89]. This leads to an overactivation and release of pro-inflammatory cytokines such as IL-2, IFN-γ and TNF-α. Each superantigen targets specific TCR Vβs, sometimes as many as seven Vβ groups, leading to a particular activation profile [90]. The associated cytokine storm results in a variety of downstream/end-organ effects and symptoms, including the potential for multi-system organ failure that can be unique for each superantigen [91]. The staphylococcal enterotoxins (SEA to SEE, SEG to SEJ, SEL to SEQ and SER to SET) are a specific subset of superantigens that have strong emetic activity. They are distinct from staphylococcal enterotoxin-like toxins (SEIK to SEIQ, SEIU to SEIX), but all are potent T-cell mitogens and are considered *S. aureus* superantigens. Over 26 superantigens have been identified, and these toxins are abundantly present across *S. aureus* clinical isolates, with most isolates having at least five superantigen genes [90,92,93,94,95]. While there are a number of staphylococcal enterotoxin-like toxins, a few have been studied more closely. SEI-X is notable because it is core-encoded and has been found in 95% of *S. aureus* strains from both humans and animals, including most USA300 strains [96,97]. SEI-X is classified not only as a superantigen, but also as a superantigen-like exoprotein (SSL) due to its sialyl-lactosamine binding site and immune evasion activities [98]. SEI-X contributes to necrotizing pneumonia in rabbits by inhibiting phagocytosis [99], although it does not reduce bacterial burden in a mouse pneumonia model [98]. Sequence studies of another enterotoxin, SEI-K, have found that at least one *seI-k* variant is conserved in nearly all USA300 strains investigated, and it is associated in particular with CA-MRSA [70,100,101,102]. SEI-K was also found to accumulate within abscesses in mice, although this was independent of the infecting *S. aureus* strain [102]. Not surprisingly, due to its close proximity on a pathogenicity island to SEI-K, SEI-Q is also expressed by most USA300 strains sequenced [97]. A study linking superantigen expression to food toxicity identified the presence of both *seIk* and *seIq* in 46% and 52% of *S. aureus* isolates, respectively [103].

In general, the clinical syndromes associated with *S. aureus* superantigens range from food poisoning to hypersensitivity or mast-cell mediated presentations, to even more severe syndromes such as toxic shock syndrome (TSS). TSS is mediated primarily by the toxic shock syndrome toxin (TSST)-1 [104]. This illness is often severe and can be fatal, with symptoms including high fever, vomiting and diarrhea, and ultimately hypotension and organ failure. Clinical studies vary in their reports of the presence of the *tsst-1* gene throughout the clinical population. Studies using PCR to detect the presence of the *tsst-1* gene have reported prevalence ranges of anywhere from ~6–68% depending on the strains studied [105,106,107,108,109]. Most isolates identified with TSS are also *mecA*-negative (MSSA strains) [27,110,111,112]. There is a strong association with *S. aureus* clonal complex 30 (CC30), with one report suggesting that 95% of the MSSA *tsst*-containing isolates belong to CC30 [112].

The enterotoxins SEA, SEB, and SEC have been widely studied for their involvement in disease. A study focusing on infective endocarditis and sepsis in rabbits found that, when rabbits were infected with strains lacking *sec*, they were less susceptible to kidney infection and death [113]. Interestingly, *S. aureus* enterotoxins have also been implicated in allergic syndromes such as atopic dermatitis (AD), a chronic inflammatory disease of the skin [114]. Over 80% of individuals with AD are also colonized with *S. aureus* in some studies [115,116], and as many as 65% of *S. aureus* strains isolated from AD patients express enterotoxins [116,117,118]. Patch tests using SEA and SEB extract performed on patients were used to confirm that these toxins may generate a hypersensitivity response leading to chronic cutaneous inflammation [116]. A number of studies measuring IgE levels to SEA and SEB during *S. aureus* infection have been performed. While these have shown conflicting results [119,120], recent studies suggest an increase in *S. aureus* colonization correlates with higher IgE serum against both SEA and SEB, leading to a more severe disease outcome [121,122,123].

As with the leukocidins, interpretation of murine models involving the staphylococcal superantigens can be challenging due to variable species tropism. This has the potential to blunt an observed effect, and, recently, transgenic mice have been developed with humanized, superantigen-responsive HLA molecules to better recapitulate the human host environment [124]. Despite these challenges, antibodies to superantigens are capable of neutralizing these toxins in several in vivo models and have been associated with protection [125]. A monoclonal antibody to SEB, 20B1, was shown to be effective against MRSA infection in multiple murine models, including sepsis, skin, and deep-tissue abscess models [126]. Passive transfer of anti-SEB antibodies developed in chickens also showed protection against lethal aerosol challenge in rhesus monkeys [127]. Similarly, a nonsuperantigenic SEB variant was cloned into a strain of *L. lactis* and tested as an oral vaccine in mice. The vaccine was capable of inducing a measurable antibody response as tested by ELISA and led to an increased survival of mice in vivo after a lethal challenge via the intraperitoneal route [128]. MAbs to SEK were also isolated and shown to enhance murine survival during infection with MRSA in a mouse toxic shock model [129].

Unique approaches to interfere against these enterotoxins have also been attempted. One group used yeast display technology to express a soluble T-cell receptor variable domain variant, L3 Vβ, capable of neutralizing both SEC and SEB in an in vitro T-cell assay. This variant was tested and found to be effective at reducing disease in four different rabbit models: LPS enhancement, miniosmotic pump, endocarditis and necrotizing pneumonia [130]. IVIg from healthy individuals have also been studied and found to successfully bind and neutralize enterotoxins and TSST by hemolysis [131]. For this reason, IVIg is often considered as an adjunct therapy for severe TSS, though efficacy data are limited [132]. Altogether, these findings suggest that antibodies to the superantigens may be capable of neutralizing and providing protection against toxin-mediated diseases in humans.

Vaccine studies against toxic shock syndrome have shown that passive transfer of TSST-1-specific antibodies can be effective at reducing death in a septic mouse model of infection [133]. This same study also used a modified TSST-1 antigen as the basis for an attenuated TSST-1 vaccine that protected mice from infection during sepsis [133]. A recombinant TSST-1 variant vaccine was later developed and has been tested for safety and tolerability in a Phase I clinical trial in Austria (NCT02340338) [134]. The vaccine was well tolerated, and a Phase II study is currently ongoing (NCT02814708). A Staphylococcal Enterotoxin B vaccine, or STEBVax, has been recently developed. STEBVax contains a recombinant form of SEB with three point mutants that prevent the toxin from binding to MHC class II. If efficacious, this vaccine would have a potential role in the prevention of SEB-mediated disease if the toxin were deployed as a bioterrorism agent, and also may have a role in a polyvalent *S. aureus* vaccine more broadly. STEBVax met all endpoints of its recent Phase I clinical trial (NCT00974935) (Figure 2) [135].

### 2.7. Exfoliative Toxins (ETs)

Exfoliative toxins (ETs) are comprised of secreted serine proteases that attack the skin by cleaving cadherins and destroying the cell–cell adhesions and junctions of the epidermis (Figure 1). The main ETs are ETA, ETB, ETC, and ETD, although ETA and ETB have been best studied due to their association with staphylococcal scalded skin syndrome (SSSS) [136]. SSSS mainly affects young children, causing extreme skin sloughing. ETA and ETB are not widely present in clinical strains, with around 4% of MSSA carrying the genes and only 10% present in MRSA isolates [137,138].

Because SSSS predominantly affects children under the age of 5, the role of antibodies in protecting children has been investigated. These studies found that preterm infants have a low antibody response when tested by ELISA, but this response increases after SSSS infection. In contrast, full-term infants, able to fully take advantage of maternal antibodies to ETA, display a higher level of anti-ETA antibodies [139,140]. The lower levels of antibodies in preterm neonates could be partially responsible for the increase in SSSS infection in this demographic [141].

## 3. Prior Attempts to Clinically Intervene against *S. aureus* Toxins

Currently, no vaccine to *S. aureus* exists, despite a plethora of unsuccessful attempts (Figure 2). Although substantial data exist to suggest a prominent role of toxins in the pathogenesis and virulence of *S. aureus*, it remains unclear which toxins are most clinically relevant. This is due to a variety of factors, including important differences in species tropism, geographical differences in commonly circulating strain types, and conflicting study designs as discussed above. Ideally, an *S. aureus* vaccine would induce a cross-neutralizing response against many toxins, which may be possible for the leukocidins given their inherent cross-reactivity. Indeed, multiple antibodies to date are able to bind and neutralize multiple leukocidins, such as ASN-1 and ASN-2 [62,85,86,87]. The PVL subunit vaccine developed by Integrated Biotherapeutics also neutralized multiple toxins, including PVL, LukED and HlgABC, but not LukAB [43,73].

An underlying problem in the development of a successful *S. aureus* vaccine, as well as a more complete understanding of the role of the leukocidins more broadly, has been the lack of consistently representative animal models [142]. Laboratory-adapted *S. aureus* strains often lack clinical relevance and may not adequately predict how effective target treatments will be in humans. On the other hand, many *S. aureus* virulence factors show species specificity or tropism, which also contributes to the problem of translation of murine models. A number of the leukotoxins (e.g., PVL, LukAB, and HlgABC) show high tropism for human over murine neutrophils. PVL, in particular, has been difficult to study as it has almost no activity in mice, with moderate species activity in rabbits. Other leukocidins, like LukED, are easier to evaluate as they target receptors in both humans and mice. For the majority of leukocidins, rabbit models can be used alongside or in place of murine models to further validate the relevance of these toxins in causing disease. As more information has come to light regarding individual toxin receptors, humanized mice can also be developed which overcome the limitation of cellular tropism [52]. Ideally, a universal humanized mouse could be developed which allows for the testing of the staphylococcal toxins more broadly.

Aside from the inherent difficulties of testing preclinical vaccines and therapeutics in animal models, there has also been a historical focus on only one side of the immune battle with phagocytes, which is opsonophagocytosis. The ability of a therapeutic to induce opsonophagocytic killing of *S. aureus* has traditionally been sufficient to warrant success in preclinical models and is the method by which most other bacterial vaccines were developed. *S. aureus,* however, is highly capable of evading opsonizing antibody responses, and is also capable of surviving within and even lysing host phagocytes.

One final strategy to effectively neutralize *S. aureus* toxins would be to target global regulators, such as the Agr and Sar pathways, using small molecules like peptides. *Agr* (accessory gene regulator) is a quorum-sensing pathway that regulates whether *S. aureus* forms a biofilm or planktonic state [143,144], but it also regulates the production of toxin genes [145,146]. Two other important global regulons that can be targeted include *sar* (staphyloccal accessory regulator) and *sae* (*S. aureus* exoprotein expression regulator). RNAIII-inhibiting peptides (RIP), have been shown to inhibit *agr* RNA transcripts and prevent staphylococcal adherence to mammalian cells, leading to a reduction in *S. aureus* pathogenesis when tested in cellulitis in vivo models [147,148]. Synthetic analogues to RIP can also be effectively produced, highlighting the possibility of these inhibitors as therapeutics.

This review describes a number of secreted virulence toxins that play a crucial role in *S. aureus* pathogenesis and allow the bacterium to attack and survive within phagocytes. Factors such as LukAB are upregulated when the bacterium is targeted by neutrophils, leading to the lysis of these cells [48,149]. By focusing on the neutralization of these toxins, a novel vaccine may warrant consideration of the important role these toxins play in targeting phagocytes, a currently under-exploited therapeutic strategy.

## 4. Conclusions

A more detailed understanding of the complicated host–pathogen interactions that occur between the toxins of *S. aureus* and their target tissues and cells will be crucial for the development of novel preventive and therapeutic measures. The broad synergy and cross-reactivity between these toxins suggest that attempts to intervene at the host–toxin level will likely require broad activity and potent anti-toxin function. More studies are needed to fully understand numerous aspects of staphylococcal toxin biology, including specific toxin roles in the face of apparent redundancy of virulence mechanisms, the differential expression or genetic presence of toxins across strain lineages, and the role of specific toxins across diverse clinical phenotypes.
